# Applied Cardio-Oncology in Hematological Malignancies: A Narrative Review

**DOI:** 10.3390/life14040524

**Published:** 2024-04-18

**Authors:** Evdokia Mandala, Kyranna Lafara, Dimitrios Kokkinovasilis, Ioannis Kalafatis, Vasiliki Koukoulitsa, Eirini Katodritou, Christos Lafaras

**Affiliations:** 1 Division of Hematology, Forth Department of Medicine, School of Medicine, Aristotle University of Thessaloniki, 54642 Thessaloniki, Greece; eudokiamandala@gmail.com (E.M.); kyralafa@gmail.com (K.L.); dkokkinovasilis@gmail.com (D.K.); 2 Cardiology-Oncology Unit, Theagenion Cancer Hospital, 54639 Thessaloniki, Greece; jkal71@gmail.com (I.K.); vkoukoul@yahoo.gr (V.K.); 3 Department of Hematology, Theagenion Cancer Hospital, 54639 Thessaloniki, Greece; eirinikatodritou@gmail.com

**Keywords:** cardio-oncology, hematological malignancies, cardiotoxicity, multimodality imaging, myocardial dysfunction

## Abstract

Applied cardio-oncology in hematological malignancies refers to the integration of cardiovascular care and management for patients with blood cancer, particularly leukemia, lymphoma, and multiple myeloma. Hematological cancer therapy-related cardiotoxicity deals with the most common cardiovascular complications of conventional chemotherapy, targeted therapy, immunotherapy, chimeric antigen receptor T (CAR-T) cell and tumor-infiltrating lymphocyte therapies, bispecific antibodies, and hematopoietic stem cell transplantation. This narrative review focuses on hematological cancer-therapy-related cardiotoxicity’s definition, risk stratification, multimodality imaging, and use of cardiac biomarkers to detect clinical and/or subclinical myocardial dysfunction and electrical instability. Moreover, the most common cardiotoxic profiles of the main drugs and/or therapeutic interventions in patients with hematological malignancies are described thoroughly.

## 1. Introduction

Applied cardio-oncology in hematological malignancies refers to the integration of cardiovascular care and oncology management for patients with blood cancers. This specialized field focuses on assessing and managing the potential cardiovascular complications that may arise during cancer treatment, particularly in hematological malignancies such as leukemia, lymphoma, and multiple myeloma. 

Cardiovascular complications can occur due to the direct effects of cancer on the heart and blood vessels, as well as the side effects of cancer treatments like chemotherapy and radiation therapy. These complications may include cardiomyopathy, arrhythmias, thromboembolic events and heart failure. In the context of applied cardio-oncology, healthcare professionals collaborate to monitor and mitigate these risks. This involves comprehensive cardiovascular assessment before and during cancer treatment, close monitoring of cardiac function, and the implementation of preventive strategies to minimize cardiovascular damage. By integrating cardio-oncology principles into the management of hematological malignancies, healthcare providers aim to optimize cancer treatment outcomes while minimizing the risk of cardiovascular complications. This multidisciplinary approach ensures that patients receive comprehensive care that addresses both their cancer and their cardiovascular health needs [1,2,3].

## 2. Risk Stratification-Hematological Cancer Therapy-Related Cardiotoxicity Definition

Risk stratification is of great value and is multifactorial, involving advanced age, pre-existing cardiovascular conditions, type and stage of hematologic malignancy, and treatment regimen. Older age is associated with an increased risk of cardiovascular complications. Patients with pre-existing heart disease, hypertension, diabetes, or other cardiovascular risk factors may be at higher risk [1,2]. Additionally, certain types and stages of hematologic malignancies may have a higher likelihood of cardiovascular complications, as the specific chemotherapeutic drugs, radiation therapy, or targeted therapies used in the treatment can influence the total risk. When refining the definition of risk stratification for hematological cancer therapy-related cardiotoxicity, it is beneficial to specify the types of hematological cancers being considered. By categorizing them according to disease entities such as leukemia, lymphoma, myeloma, and others, the risk assessment can be more targeted and tailored to the specific characteristics and treatment regimens associated with each type of cancer. Each type of hematological cancer has distinct features in terms of pathophysiology, prognosis, treatment options, and potential cardiotoxic effects from therapy. In the treatment of hematologic malignancies, several classes of drugs are commonly used to target cancer cells and inhibit their growth. These drugs include conventional chemotherapy agents such as anthracyclines (e.g., doxorubicin), alkylating agents (e.g., cyclophosphamide), and antimetabolites (e.g., cytarabine). Tyrosine kinase inhibitors (e.g., imatinib, dasatinib, nilotinib) and monoclonal antibodies (e.g., rituximab, daratumumab) are examples of targeted therapies used in certain hematologic malignancies. Finally, types of immunotherapy such as immune checkpoint inhibitors (e.g., pembrolizumab, nivolumab), chimeric antigen receptor (CAR)-T cell therapy and hemopoietic stem cell transplantation are emerging treatments for hematologic malignancies [4,5]. Healthcare providers need to consider these risk factors and cardiotoxic profiles when designing treatment plans for patients with hematologic malignancies. Regular monitoring of cardiac function and close collaboration between hematologists and cardiologists are crucial in order to minimize the risk of cardiovascular complications and ensure optimal patient care.

Hematologic cancer therapy-related cardiotoxicity refers to the adverse effects on the cardiovascular system that can occur as a result of treatment for hematologic malignancies, such as leukemia, lymphoma, and multiple myeloma. Various treatment modalities used in hematologic cancer therapy, including conventional chemotherapy, targeted therapies, immunotherapies and radiation therapy, can potentially lead to a range of cardiovascular complications. The development of cardiotoxicity during hematologic cancer therapy can have significant implications for patient outcomes, as it may necessitate dose reductions or the discontinuation of potentially life-saving treatments. Therefore, it is crucial to identify and monitor cardiotoxicity early on, allowing for timely intervention and management; the implementation of strategies to mitigate further damage; the adjustment of treatment plans; and the provision of appropriate supportive care to minimize the impact on the patient’s cardiovascular health [2,5,6]. 

Fortunately, there has been a universal agreement on hematological-related cardiovascular toxicities’ definition, which may have important implications in the diagnosis, treatment, and follow-up of these patients, as well as for future research and clinical trials. These include the following: A. Symptomatic hematological cancer therapy-related cardiac dysfunction (HCTRCD), characterized by heart failure syndrome, classified as very severe (HF requiring inotropic and/or mechanical circulatory support), severe (HF needing hospitalization), moderate (outpatient intensification of HF therapy) or mild (stable HF treatment).

B. Asymptomatic HCTRCD, based on threshold changes of LVEF detected through echocardiographic study during cancer treatment or as an incidental finding during surveillance, including more sensitive methods to detect and confirm cardiac dysfunction, such as the new relative decline in global longitudinal strain (GLS) and the concomitant use of cardiac serum biomarkers-cardiac troponin (cTn) I or T and natriuretic peptides (NP) [2,3]. 

Additionally, the most common clinical manifestations of cardiovascular toxicity include myocarditis (immune-related, especially after extensive use of checkpoint inhibitors); vascular toxicity (either asymptomatic or symptomatic, as venous or arterial thrombosis, stroke, ACS, PAD, vasospastic and/or microvascular angina, or carotid artery disease); arterial hypertension; and cardiac arrhythmias (bradycardias, supraventricular tachycardias, ventricular arrhythmias, atrial fibrillation, torsade de pointes) [1,3,7]. 

## 3. Cardiac Imaging Modalities and Cardiac Biomarkers in Patients with Hematologic Malignances

Echocardiography is the cornerstone low-cost modality for the screening, surveillance, and detection of hematological cancer therapy-related cardiac dysfunction (HCTRCD) including two-dimensional (2D) and three-dimensional (3D) volumes, 2D and 3D LV ejection fraction (LVEF), global longitudinal strain (GLS), right ventricular (RV) size and systolic function assessment. 2D volumetric Simpson’s is recommended, preferably from measurements in apical four- and two-chamber views. As diastolic dysfunction can precede systolic dysfunction, full diastolic assessment should be done at the baseline and all sequential imaging. When two adjacent LV segments from any apical view are not adequately seen, contrast is recommended, and should then be used in all sequential testing [2,3,8,9]. 

It should be emphasized that 3D LVEF reportedly has superior reproducibility compared to 2D LVEF. However, it remains very sensitive to image quality and has low availability in real-world clinical practice. GLS through speckle-tracking echocardiography detects myocardial deformation from base to apex, as it refers to the change in length of the myocardium between relaxation and contraction, quantified by dedicated speckle tracking software which tracks different regions of the myocardium based on their unique speckle patterns, with more negative values indicating greater deformation [2,9,10].

CMR, as a second-line imaging modality, should be considered for the assessment of biventricular cardiac function when echocardiography is unavailable or non-diagnostic, due to poor-quality echocardiographic windows. Moreover, CMR can depict structural changes in the myocardium, including signs of edema and inflammation, before LV dysfunction. On the other hand, the higher cost, adverse reactions to gadolinium contrast agents, contraindications related to renal failure, the presence of metal devices and longer scanning times causing claustrophobia are the main advantages of the above imaging approach [2,3,11].

Multigated acquisition nuclear imaging (MUGA) should be considered as a third-line modality due to radiation exposure when TTE and CMR are both unavailable or non-diagnostic. Nuclear medicine can provide important insights into the early detection of impending cardiotoxicity, assist in the monitoring of cardiotoxic therapy, and may also be used as a tracking tool in the investigation of cardiotoxicity in novel therapies. SPECT MPI has a very low intra- and inter-observer variability; it allows accurate assessment of regional and global wall motion, phase analysis, and ventricular volumes in a single examination without the examination protocol having to be extended; and its value is validated by studies involving thousands of patients [11,12].

PET allows us to image changes in cellular metabolism with high sensitivity and therefore, appears well suited to identifying earlier stages of cardiomyocyte toxicity before irreversible myocardial damage develops. It should be established that cardiotoxic therapy may cause abnormalities in myocardial glucose metabolism. Additionally, myocardial inflammatory reactions may occur as a result of cancer therapy detected by PET, when the patient is adequately prepared. Also, 2-[18F]FDG PET is useful in detecting altered metabolism of the myocardium or in detecting cardiac inflammatory effects. Finally, cardiac perfusion PET may be applied for the effective detection of myocardial ischemia in patients who are candidates for potentially cardiotoxic cancer treatments or as a late sequelae after therapy, to monitor myocardial blood flow (MBF) or myocardial flow reserve (MFR) and cardiotoxic therapy or to explore mechanisms of cardiotoxicity [11,13,14,15,16].

The application of serum cardiac biomarkers to detect cardiotoxicity at a stage before it becomes irreversible is well known. The most important markers of cardiac injury are cardiac troponins and natriuretic peptides, whilst markers of inflammation such as interleukin-6, C-reactive protein, myeloperoxidase, Galectin-3 and growth differentiation factor-15, micro-ribonucleic acids, and immunoglobulin E are under investigation. Cardiac troponins (cTn) (particularly troponin I) are the gold standard biomarkers for the detection of cardiac injury and cardiomyocyte necrosis, and the most extensively used biomarkers to detect cardiac toxicity. Moreover, brain natriuretic peptide (BNP) and N-terminal pro-B type natriuretic peptide (NT-proBNP) are simulated to be secreted by cardiomyocytes from increased transmural tension and neurohormonal stimulation operating as sensitive indicators of pressure overload, myocardial stretch, and eventually cancer therapy-related cardiac dysfunction. Cardiac serum biomarkers are widely used for risk stratification at the baseline and during therapy, although recommendations concerning therapeutic strategies are mostly based on expert opinion. Baseline cardiac serum biomarker measurements are required if the degree of change in the biomarkers is to be used to identify subclinical cardiac injury during cancer treatment or to identify high-risk patients. There is a need for larger prospective studies to validate the clinical utility of the emerging serum biomarkers in the risk stratification and modification of treatment strategies to mitigate adverse cardiovascular events in patients with hematological malignancies [17,18,19].

## 4. Main Anticancer Drugs in Hematological Malignances and Cardiotoxic Profiles

The treatment of lymphomas consists of chemotherapy either alone or in combination with radiotherapy. It is well known that the standard treatment for Hodgkin lymphoma is ABVD (Adriamycin, Bleomycin, Vinblastine, and Dacarbazine), but other regimens such as the Stanford V (doxorubicin, vinblastine, mechlorethamine, etoposide, vincristine, bleomycin, and prednisone) and escalated-BEACOPP (Bleomycin, Etoposide, Adriamycin, Cyclophosphamide, Vincristine, Procarbazine, and Prednisone) treatments can be used. Additionally, R-CHOP (Rituximab, Hydroxydoxorubicin, Vincristine [Oncovin], Prednisolone), administered every 3 weeks for up to six cycles with curative intent, is the main chemotherapeutic regimen for most types of non-Hodgkin lymphomas [20]. 

## 5. Conventional Chemotherapy’s Related Cardiovascular Toxicities

### 5.1. Anthracyclines

It is well known that anthracyclines represent the cornerstone conventional chemotherapeutic agents in hematologic malignancies. Anthracycline-induced cardiotoxicity is potentially a continuous dose-dependent phenomenon causing immediate myocardial cell injury, myocardial deformation, asymptomatic cardiotoxicity, and finally, overt cardiotoxicity and consequently heart failure syndrome [21,22,23]. Identification of both symptomatic and asymptomatic HCTRCD should be achieved by careful clinical assessment, estimation of serum cardiac biomarkers, and cardiac imaging using the classic and modern modalities of transthoracic echocardiography. It is crucial to emphasize the importance of patients’ risk stratification, as high-risk oncologic patients should undergo, in addition to thorough baseline screening, echocardiography every second cycle of chemotherapy, as well as estimation of biomarkers, troponin, and/or natriuretic peptides during each visit [2,3,6]. Primary prevention strategies include CV factors’ control, limitation of AC doses, use of less cardiotoxic AC analogs (liposomal and/or pegylated AC), Dexrazoxane, and concomitant use of b-blockers, RAS inhibitors, and statins in high-risk patients. There is an anthracycline equivalence dose when doxorubicin is considered as a reference of a cumulative dose above 400 mg/m^2^, epirubicin above 900 mg/m^2^, daunorubicin > 900 mg/m^2^ and idarubicin > 150 mg/m^2^, as the incidence of HF rises > 5% [2,23,24,25].

### 5.2. Bleomycin

Cardiovascular toxicity is a rare adverse effect of bleomycin and may be expressed clinically as hypotension, pericarditis, acute substernal chest pain, coronary artery disease, myocardial ischemia, myocardial infarction, and cerebral vascular accident, with an incidence of 1–3% [26,27]. 

### 5.3. Vincristine

The main cardiovascular side effects provoked by vincristine are myocardial ischemia and infarction, which tend to occur during or shortly after therapy and might, therefore, be related to coronary artery vasospasm as a result of cellular hypoxia, causing significant transitory impairment in the vasodilator and the contractile function, as well as temporary vascular damage [27].

### 5.4. Cyclophosphamide 

Cyclophosphamide (CP) is a nitrogen mustard alkylating agent with potent antineoplastic, immunosuppressive, and immunomodulatory properties. It and its metabolites aldophosphamide, 4-hydroxy cyclophosphamide, and acrolein are identified as cardiotoxic agents, with the last being the most toxic metabolite. The proposed molecular mechanisms of CP cardiotoxicity include increased oxidative and nitrosative stress; cardiac calcium overload; reduced mitochondrial fatty acid oxidation; myocardial inflammation; apoptosis; and endothelial damage, leading to myocardial systolic and diastolic dysfunction. The incidence of fulminant congestive heart failure is reported to be 5% to 19%. Symptoms may include arrhythmias, acute fulminant heart failure, myopericarditis, pericardial effusion, and cardiac tamponade, usually occurring within 1 to 3 weeks. Older age, previous or concomitant use of anthracyclines, and prior mediastinal irradiation are the main risk factors for drug-induced cardiotoxicity [28,29].

## 6. Targeted Therapy’s Related Cardiovascular Toxicities

### 6.1. Rituximab

Rituximab is a monoclonal antibody against the CD20 antigen on malignant B lymphocytes, widely used to treat a variety of hematologic malignancies. It may cause angina pectoris, acute coronary syndromes, cardiac arrhythmias, hypertension and/or hypotension, heart failure, and Takotsubo cardiomyopathy, particularly as acute reactions related to the manifestation of cytokine release syndrome. These acute infusion-related effects have been reported during less than 1 percent of infusions [26,30].

### 6.2. Breakpoint Cluster Region–Abelson Oncogene Locus Tyrosine Kinase Inhibitors

It is well known that chronic myeloid leukemia (CML) is associated with chromosomal translocation (i.e., Philadelphia chromosome), which encodes the BCR: ABL1 oncoprotein through the fusion of the BCR and ABL1 genes with active tyrosine kinase activity, so tyrosine kinase inhibitors (TKIs) are currently the cornerstone treatment of CML. The cardiovascular toxicities of small-molecule TKIs targeting BCR-ABL, imatinib, dasatinib, nilotinib, bosutinib, and ponatinib are due to their ‘on- and off-target’ effects. Furthermore, there is individualized variability in cardiotoxicity as a result of TKI targets, genetic predisposition, and cardiovascular risk factor interactions. The 1st generation BCR-ABL TKI imatinib causes in 0.1–1% of cases hypertension, heart failure, hyperglycemia, and pleural effusion, while 2nd generation nolitinib causes hypertension, QTc prolongation, atrial fibrillation, dyslipidemia, and vascular toxicity (1–10%). Dasatinib is commonly responsible for pleural effusion (10%), while less common adverse drug reactions include hypertension, heart failure, pericardial effusion, and pulmonary hypertension (1–10%). The last-generation BCR-ABL TKI bosutinib may cause hypertension, as well as pleural and pericardial effusion, while ponatinib, 3rd generation BCR-ABL TKI may cause hypertension (10%), atrial fibrillation, heart failure, hyperglycemia, dyslipidemia, pericardial or pleural effusion, and vascular toxicity (1–10%). Baseline risk assessment and monitoring and baseline echocardiography during the usage of first- and third-generation tyrosine kinase inhibitors, and then every 3 months during the first year and every 6 months thereafter, is recommended. Moreover, QTc estimation should be considered at the baseline, 2–4 weeks after TKI’s administration, and 2 weeks after any dose increase (IIa, C), while echocardiography should be performed every 3 months for high-risk patients (IIa, C) [31,32,33].

### 6.3. Bruton Tyrosine Kinase (BTK) Inhibitors

BTK inhibitors (Ibrutinib, Acalabrutinib, Zanubrutinib, Pirtobrutinib) represent a promising therapeutic target for various B cell malignancies (chronic lymphocytic leukemia, small lymphocytic lymphoma, marginal zone lymphoma, diffuse large B cell lymphoma, and follicular lymphoma), Waldenstrom macroglobulinemia, and multiple myeloma. Their main cardiotoxic effects include atrial fibrillation and flutter, hypertension, ventricular arrhythmias, and sudden cardiac death [34,35]. The mechanism of arrhythmia from BTK inhibitors involves off-target inhibition of Tec protein tyrosine kinase (TEC) and downstream phosphoinositide 3-kinase (Akt) signaling, leading to enhanced automaticity, prolonging cardiac action potential which increases vulnerability to early and delayed afterdepolarizations. The cardiotoxicity linked to BTK inhibitors stems from their interference with signaling pathways that are essential for cardiac function. While the primary target of BTK inhibitors is the B-cell receptor signaling pathway in cancer cells, these drugs can also affect other kinases and signaling molecules in the heart, leading to adverse cardiac events. In conclusion, hematological cancer therapy-related cardiotoxicity associated with BTK inhibitors is a class effect driven by the drugs’ mechanisms of action. Second-generation BTK inhibitors like acalabrutinib and zanubrutinib offer improved cardiotoxicity profiles due to their enhanced selectivity and reduced off-target effects on cardiac tissues [36,37]. Particularly, the administration of Ibrutinib is associated with AF (13–16%) initiated in the first 3 months, which may persist despite stopping or reducing the dose. Moreover, BTK inhibitors especially Ibrutinib are responsible for an approximately 50% risk of bleeding, as a result of the inhibition of multiple pathways that regulate platelet function. Although anticoagulation is still recommended for CHA2DS2-VASc>2 if there is not a high risk of bleeding, optimal management involves multidisciplinary collaboration between cardio-oncologists and hematologists [35,38].

## 7. Multiple Myeloma Therapeutic Regimes

Therapeutic regimes for multiple myeloma include alkylating agents (cyclophosphamide, melphalan), proteasome inhibitors (bortezomib, carfilzomib, and ixazomib), immunomodulatory drugs (thalidomide, lenalidomide, pomalidomide) and monoclonal antibodies (daratumumab, elotuzumab, and isatuximab). The most popular regiments are bortezomib, lenalidomide, dexamethasone (“VRd”) and daratumumab, lenalidomide, dexamethasone (“DRd”). Evaluation of patients’ eligibility for stem cell transplantation is crucial, as it offers a significant and prolonged response. If they are candidates for autologous stem cell transplantation, they are often treated with a four-drug regimen such as DVRd or DVTd, followed by lenalidomide in combination with a proteasome inhibitor, or by daratumumab as maintenance therapy. Bispecific antibodies (teclistamab, elranatamab, and talquetamab) are designed to simultaneously bind to a target moiety on tumor cells and to CD3 on T cells, then activate the patient’s T cells to kill their tumor cells, and have shown impressive results in relapsed refractory myeloma. Additionally, the nuclear export inhibitor, selinexor, can be used for the treatment of multiply relapsed multiple myeloma. A modern therapeutic intervention is CAR-T cell therapy, a form of immunotherapy including the patient’s immune cells (T cells) which are collected, genetically modified, and re-induced, directly targeting the cancer cells of the patient [39,40,41].

## 8. Multiple Myeloma Drug-Related Cardiovascular Toxicities

Alkylating agents may cause heart failure and atrial fibrillation (1–10%), and immunomodulatory drugs may cause venous and arterial thromboembolic events (0.1–10%), hypertension (1–10%), hyperglycemia (1–10%), and atrial fibrillation (0.1–10%). Additionally, the main adverse cardiovascular events of proteasome inhibitors, particularly carfilzomib, including heart failure, hypertension, ischemia, and atrial fibrillation (>18%) usually occur during the first three months of therapy and are more common among patients with elevated baseline levels of brain natriuretic peptide (BNP) or NT-proBNP [37,39]. As the increase in venous thromboembolism (VTE) takes place, thromboprophylaxis is advised when carfilzomib is administered in combination with lenalidomide and dexamethasone. Monoclonal antibodies are associated with an increased risk of hypertension, diabetes mellitus, heart failure, atrial fibrillation, and venous thrombotic events (1–10%). Cardiovascular monitoring in patients with multiple myeloma receiving all the therapeutic regimens is of great value both at the baseline and during treatment. In particular, all myeloma patients should undergo thorough clinical assessment, BP, ECG, TTE, NT-Pro-BNP, and cTn at the baseline and clinical evaluation; BP during every clinical visit, NP and cTn every cycle during the first six cycles; and TTE every three cycles under carfilzomib or bortezomib, particularly in high-risk patients [2,3,42].

It is well known that thrombogenicity in MM is multifactorial, as a result of a combination of patient, disease, and treatment-related factors. Patient-related factors include advanced age; history of venous thromboembolism; obesity; immobility; central venous catheter; acute infection or hospitalization; comorbidities; history of inherited thrombophilia; recent surgery; and ongoing hormone therapy. Disease-related factors include the stage of active multiple myeloma; evidence of hyperviscosity; pathological fractures conditioning immobilization; and the requirement of surgery. Finally, treatment-related factors include immunomodulatory drugs in combination with high-dose dexamethasone; multi-agent chemotherapeutic regimens; and/or exposure to erythropoietin-stimulating agents. Thus, thromboprophylaxis with LMWH is recommended for patients with MM, at least during the first 6 months of therapy (Class I, Level A), and therapeutic doses of LMWH are recommended for MM patients with previous VTE (Class I, Level B) [2,43]. 

In the modern era of DOACs, thromboprophylaxis with low doses of xabans (apixaban, edoxaban, rivaroxaban) should be considered as an alternative to LMWH (standard of care), at least during the first 6 months of therapy. The drug-drug interactions of antithrombotic agents and antimyeloma drugs, as well as patients’ compliance and preferences, should be taken into account in the choice of thromboprophylaxis. Severe thrombocytopenia (platelet count < 20 × 10^9^/L), active bleeding, congenital bleeding disorders (hemophilia, von Willebrand disease, severe deficiency of coagulation factors), and acquired coagulopathy are absolute contraindications to thromboprophylaxis [2,3,43,44].

Historically, AL amyloidosis was considered a rare metabolic disorder due to challenges in its diagnosis and limited awareness among healthcare professionals. However, with the advent of systematic screening programs and the establishment of specialized cardiac amyloid clinics, the perception of AL amyloidosis as a rare disease has shifted. Insoluble fibrils are deposited in various tissues, causing organ dysfunction and eventually death; therefore, early detection is crucial. AL amyloidosis is most commonly diagnosed when the affected patient has less than 10% bone marrow plasma cells the quantity required to make a diagnosis of myeloma. Patients with a higher burden of disease, as manifested by high levels of free light chains, increased numbers of bone marrow plasma cells, and more severe end-organ involvement, are more likely to experience treatment failure and worse prognosis. Amyloid deposits may result in a wide range of clinical manifestations depending upon their type, location, and amount in various organs. Thus, the most common clinical manifestations include gastrointestinal disease (hepatomegaly with or without splenomegaly, bleeding, gastroparesis, constipation, bacterial overgrowth, malabsorption, and intestinal pseudo-obstruction), renal involvement (asymptomatic proteinuria or clinically apparent nephrotic syndrome), peripheral and autonomic neuropathy, central nervous system disease (cerebral amyloid angiopathy, cortical and subcortical intracranial bleeding, ischemic embolic stroke), cardiac involvement (infiltrative cardiomyopathy), skin manifestations (waxy thickening, ecchymoses, subcutaneous nodules or plaques, raccoon eyes), and pulmonary disease (tracheobronchial infiltration, pleural effusions, amyloidomas, and pulmonary hypertension) [45,46]. Finally, the diagnostic evaluation is based on the clinical presentation of amyloidosis. Cardiac amyloidosis is presented as heart failure, angina, syncope/presyncope, unexplained “thickening of left and/or right walls”, ECG abnormalities (low voltage, conduction disease), pericardial disease, and thromboembolism/stroke. Therapy is individualized and should be risk-adapted and response-tailored cardiac biomarkers (NT-Pro-BNP, cTn). Chemotherapeutic regimens are the cornerstone therapy including alkylating agents (melphalan, cyclophosphamide), proteasome inhibitors (bortezomib, ixazomib), monoclonal antibodies (daratumumab), and anti-myeloid antibodies (NEOD001). The aim of therapy is a clonal hematologic response, a cardiac response, and improved survival. Treatment should be guided by the ASCT eligibility (~20%) of patients, given the robust and durable response, and preceded by induction chemotherapy with bortezomib-based regimens [2,45,47].

## 9. Chimeric Antigen Receptor T (CAR-T) Cell and Tumor-Infiltrating Lymphocytes Therapies

**Figure 1:**
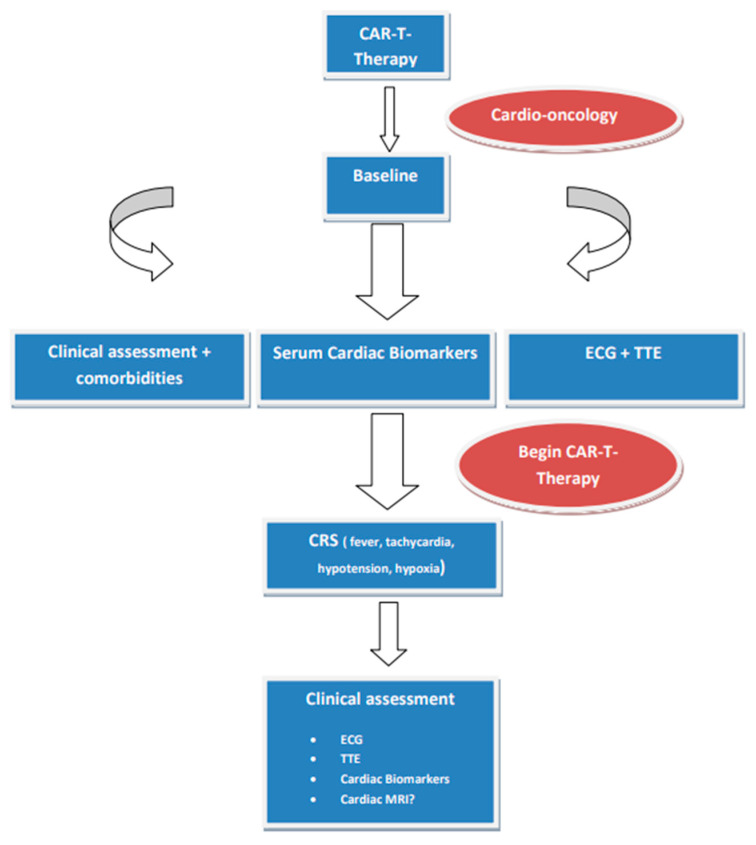
Proposed protocol in patients treated with CAR-T cells. Abbreviations: CRS, cytokine release syndrome; ECG, electrocardiogram; MRI, magnetic resonance imaging; TTE, transthoracic echocardiogram.

CAR-T cells, used as a form of genetically modified autologous immunotherapy, are genetically modified ex vivo and infused back into the patient, encoding a chimeric antigen receptor to direct the patient’s T cells against the leukemic cells. This modern immunotherapy is currently used for the treatment of acute lymphocytic leukemia and aggressive B-cell lymphomas when they are refractory or in second or later relapse, as well as for the treatment of relapsed and refractory multiple myeloma [48,49,50]. Cardiovascular toxicities are associated with the occurrence of cytokine release syndrome (CRS), manifesting as left ventricular dysfunction, heart failure, cardiac arrhythmias, pericardial effusion, Takotsubo cardiomyopathy, and cardiac arrest (Figure 1). Additional adverse events are hypersensitivity reactions, infections, prolonged cytopenias, hypogammaglobulinemia, and second malignancies. The degrees of CAR-T cell activation and activation kinetics are influenced by the level of tumor antigen expressed on malignant cells, tumor burden, the antigen binding domain’s affinity to its target epitope, and the CAR’s costimulatory elements [51]. It should be emphasized that CRS usually responds to treatment with the Interleukin-6 receptor antagonist tocilizumab, administered with or without glucocorticoids [49,50,52].

## 10. Bispecific Antibodies

Bispecific T-cell engagers represent a new class of immunotherapy that induces the patients’ immune cells to attack tumors by retargeting T-cells to tumor cells. These bispecific antibodies are currently being investigated in DLBCL (mosunetuzumab, glofitamab, epcoritamab, and odronextamab) in intensively treated patients after CAR-T cell therapy, particularly in patients who will not achieve a durable remission. Bispecifics have lower rates of CRS compared with CAR-T, although there are limited data in patients with underlying heart disease. Cardiovascular side effects include hypotension (24–31%), tachycardia (24–31%), and atrial fibrillation (5%) [53,54]. Teclistamab-cqyv is a humanized antibody and a bispecific BCMA-directed CD3 T-cell engager used for the treatment of patients with relapsed or refractory multiple myeloma. BCMA is a protein expressed by late-stage B cells and plasma cells. Treatment-related adverse events have occurred, particularly CRS and ICANS (immune effector cell-associated neurotoxicity syndrome), but less frequently and less severely compared with CAR T-cell therapy. Cardiovascular complications are due to CRS: hypertension (18%), hypotension (12%), and cardiac arrhythmias (16%) [55,56]. 

## 11. Hematopoietic Stem Cell Transplantation 

Patients with hematological relapsed and/or refractory malignancies are suitable for hematopoietic stem cell transplantation. A graft of hematopoietic stem cells is infused to restore hematopoiesis following the administration of conventional therapy (intensive chemotherapy and/or radiation). The graft can be the patient’s cells (autologous HCT), or the donor can be another individual (allogeneic HCT). Cardiovascular surveillance in patients in patients referred for hematopoietic stem cell transplantation (HSCT) includes baseline CV assessment, blood pressure measurement, ECG, lipid profile, HbA1C, and echocardiography. Risk stratification is of great value, as high-risk patients (allogenic HSCT, pre-existing heart disease and co-morbidities, and oncologic treatment history) should undergo a thorough clinical evaluation, TTE, NP and ECG 3 and 12 months after HSCT if new cardiac symptoms occur at any time and annually thereafter. It should be emphasized that HSCT is associated with excess cardiovascular risk partially due to exposure to cardiotoxic chemotherapy and radiation, as well as indirect and direct detrimental effects on cardiovascular reserve [2,57,58].

## 12. Radiotherapy

The toxic effects of mediastinal radiotherapy on heart substructures, such as the pericardium, myocardium, conducting system, coronary arteries, and heart valves, are well documented. Most of our knowledge regarding cardiotoxicity in long-term Hodgkin and non-Hodgkin lymphoma survivors comes from radiation therapy delivered in the past using mantle field and prescribed radiation doses of ≥40 Gy [57,58,59]. It is well known that the cardiotoxicity of radiotherapy is multifactorial, as cumulative radiation dose, irradiated heart volume, young age at the time of radiation exposure (especially doses > 30–50 Gy), fractional dosage (>2 Gy), the time elapsed since exposure, concomitant use of other chemotherapeutic agents, and classical cardiovascular risk factors play a pivotal role. Fortunately, the development of radioprotective oncology has led to the minimization of mean heart dose, and more targeted application of dosage, which reduces the amount of irradiated heart tissue. Additionally, modern heart-sparing RT strategies such as the optimal use of modern intensity, modulated photon RT technologies, the use of deep inspiration breath-hold or respiratory-gated techniques, image-guided RT, and personalized management prevent and attenuate CV complications [2,60,61]. Table 1 summarizes the main therapeutic interventions, risk factors, indications, mechanisms of cardiotoxicity, and HCTRC in hematological malignancies.

## 13. Conclusions and Future Directions

**Figure 2:**
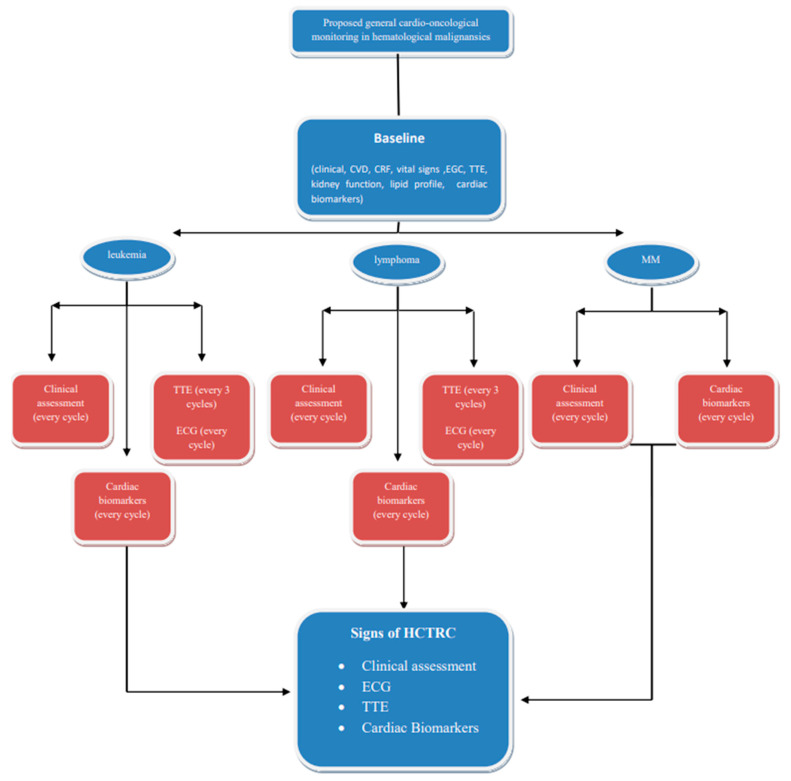
Proposed simplified cardio-oncological monitoring/surveillance algorithm in hematological malignancies. This protocol should be modified according the possible HCTRC effects of therapeutic regimens. Abbreviations: CVD, cardiovascular disease; CRF, cardiac risk factors; ECG, electrocardiogram; HCTRC, hematological cancer therapy-related cardiotoxicity; MM, multiple myeloma; TTE, transthoracic echocardiogram.

Patients with hematological malignancies are vulnerable to cardiovascular complications due to the type and stage of the disease, as well as patient and/or therapy-related risk factors that adversely impact survival. Comprehensive cardiac evaluation before, during, and after treatment is crucial, and the incorporation of surveillance strategies leads to early detection and management, resulting in improved survival via the multidisciplinary interactions of hematologists and cardio-oncologists for optimal medical care. Risk stratification is of great value as close monitoring is suggested for high-risk hematological cancer patients who are recommended to receive severe cardiotoxic therapy. Adopting a disease-specific approach to risk stratification for hematological cancer therapy-related cardiotoxicity aligns with the principles of precision medicine. This personalized approach allows for more accurate risk assessment and proactive management strategies that are tailored to each patient’s unique clinical profile and treatment plan (Figure 2). The early detection, diagnosis, and treatment of therapy-related CV complications are carried out according to ESC guidelines on cardio-oncology, developed in collaboration with the European Hematology Association (EHA); the European Society for Therapeutic Radiology and Oncology (ESTRO); and the International Cardio-Oncology Society (IC-OS) developed by the task force on cardio-oncology of the European Society of Cardiology. Furthermore, it is essential to record cardio-hematology registries in order to collect ‘big data’, a prerequisite for artificial intelligence to identify and predict the risk of HCTR-CVT and responses to specific cardioprotective interventions.

## Figures and Tables

**Table 1:** The main therapeutic interventions, risk factors, indications, mechanisms of cardiotoxicity, and HCTRC in hematological malignancies.

Therapeutic Interventions	Drug Class	Drugs	Risk Factors	Indications	Mechanisms of Cardiotoxicity	HCTRC
Conventional chemotherapy						
	Anthracyclines	Doxorubicin	Age (<5 or >65)	Leukemias	ROS	Subclinical myocardial dysfunction
		Epirubicin	Female sex	Lymphomas	DNA damage	HF
		Daunorubicin	Hypertension		Apoptosis	Arrythmias
		Idarubicin	DM		miRNAs	
			AF		Fibrosis	
			CAD			
			Prior mediasternal RT			
			Prior cardiotoxic cancer treatment			
			Cumulative dose doxorubicin ≥ 250 mg/m^2^ or equivalent			
			Genetic factors			
	Antibiotics					
		Bleomycin				
			Age	Hodgkin and non-Hodgkin Lymphomas	Oxidative stress	Hypotension
			Cardiac disease		Mitochondrial dysfunction	Pericarditis
			Dose		Impaired energy metabolism	Myocardial ischemia
						Stroke
	Vinca Alkaloids					
		Vincristine				
				ALL	Coronary artery vasospasm	Myocardial ischemia and infarction
				Burkitt Lymphomas	Cellular hypoxia	Arrythmias
				Hodgkin and non-Hodgkin Lymphomas	Endothelial dysfunction	Myocardial dysfunction
				MM		
	Alkylating agents					
		Cyclophosphamide	Age	Lymphomas	Oxidative and nitrosative stress	HF
			Prior mediastinal RT	MM	Cardiac calcium overload	Myopericarditis
			Anthracyclines	Leukemias	Mitochondrial damage	Arrythmias
					Endothelial dysfunction	Tamponade
					Myocardial inflammation	
Targeted therapy						
	BCR-ABL1 TKIs					
		Omatinib	Genetic predisposition	CML	On- and off-target effect	Hypertension
		Dasatinib	CRF	Ph+ ALL	Mitochondrial damage and dysfunction	Hyperglycemia
		Nilotinib			ROS	Arrythmias/QTc prolongation
		Bosutinib			Caspase activation	Vascular Toxicity
		Ponatinib			Apoptosis	Dyslipidemia
						Pleural and pericardial effusion
						Myocarditis/Pericarditis
						HF
						Stroke
						ACS
						VTEs
	BTKIs					
		Ibrutinib	Age	CLL	Off-target effect	Arrythmias/AF
		Acalabrutinib	Hypetension	Lymphomas	Early and delayed afterdepolarizations	Hypertension
		Zanubrutinib	Hyperlipidemia	WM	Platelet dysfuntion	SCD
		Pirtobrutinib	DM	MM		CNS hemorrhage
			Structural heart disease			Stroke
						Myocardial dysfunction
	Proteasome inhibitors					
		Bortezomib	Age	MM	Mitochondrial dysfunction	Hypertension
		Carfilzomib	Coexisting cardiac disease	MCL	Reduction of ATP synthesis	Myocardial dysfunction
			Renal failure		Myocardial damage	HF
			MM-associated comorbidities			Thrombosis
						Arrythmias
	Immunomodulatory drugs					
		Thalidomide	MM-associated comorbidities	MM	Anti-TNF	Arrythmias
		Lenalidomide	Concommitant PI		Anti-angiogenic effects	VTEs
		Pomalidomide			Endothelial dysfunction	MI
					Proteasome mediated protein degradation	Stroke
					Cereblon activation	HF
	Immunotherapy					
		Rituximab	Age	NHL	CRS	Angina
			Coexisting cardiac disease	CLL	Platelet dysfuntion	ACS
			Prior radiation therapy			Takotsubo
			Tumor lysis syndrome			Hypotension/Hypertension
			Cumulative dose			HF
			Hypersensitivity			Arrythmias
	CAR-T cell therapy		Age	ALL	CRS	Myocardial dysfunction
			Tumor burden	Aggressive B-cell lymphomas	Off-target effect	HF
			Coexisting cardiac disease	Refractory MM	Cross-reactivity between T-cells and titin	Takotsubo
			Autoimmune disease			Arrythmias
			Genetic factors			Pericardial effusion
						SCD
						Leak syndrome
	Bispecific antibodies	Mosunetuzumab	Age	DLBCL	CRS	Hypotension/Hypertension
		Glofitamab	Coexisting cardiac disease	Refractory MM	Off-target effect	Arrythmias/AF
		Odronextamab	Dose	AML	Immunogenicity	
		Teclistamab-cqyv	Prior cardiotoxic cancer treatment			
Hematopoietic stem cell transplantation			Allogenic HSCT	Leukemias	GVHD	Arrythmias/AF
			Coexisting cardiac disease	Lymphomas	Endothelial dysfunction	HF
			Cardiotoxic cancer treatment	MM		
			RT			
Radiotherapy			Younger age	Hodgkin and non-Hodgkin Lymphomas	Oxidatve stress	CAD
			Cumulative radiation dose		ROS	VHD
			Smoking		Mitochondrial dysfunction	Pericardial disease
			Prior cardiotoxic cancer treatment		Cytoplasmic calcium overload	HF
			Dose per fraction (>2 Gy/day)		ncRNAs	Myocardial fibrosis
			Techniques of RT		Endothelial dysfunction	Conduction system disease
					Inflammation	
					Fibrosis	
					Autonomic dysfuntion	

Abbreviations: ACS, acute coronary syndrome; AF, atrial fibrillation; ALL, acute lymphoblastic leukemia; AML, acute myeloid leukemia; anti-TNF, anti-tumor necrosis factor; BTKIs, bruton tyrosine kinase inhibitors; CAD, coronary artery disease; CLL, chronic lymphocytic leukemia; CNS, central nervous system; CRF, cardiac risk factors; CRS, cytokine release syndrome; DLBCL, diffuse large B cell lymphoma; DM, diabetes mellitus; GVHD, graft versus host disease; HCTRC, hematological cancer therapy-related cardiotoxicity; HF, heart failure; HSCT, hematopoietic stem cell transplantation; MCL, mantle cell lymphoma; MI, myocardial infarction; MM, multiple myeloma; ncRNAs, non-coding RNAs; NHL, non-Hodgkin lymphoma; Ph+ ALL, Philadelphia chromosome-positive ALL; PIs, proteasome inhibitors; ROS, reactive oxygen species; RT, radiotherapy; SCD, sudden cardiac death; VHD, valvular heart disease; VTEs, venous thromboembolic events; WM, Waldenstrom’s macroglobulinemia.
